# The Prognostic Relevance of MRI Characteristics in Myxofibrosarcoma Patients Treated with Neoadjuvant Radiotherapy

**DOI:** 10.3390/cancers15102843

**Published:** 2023-05-19

**Authors:** Stefan G. van Ravensteijn, Maikel J. L. Nederkoorn, Tom C. P. Wal, Yvonne M. H. Versleijen-Jonkers, Pètra M. Braam, Uta E. Flucke, Johannes J. Bonenkamp, Bart H. W. Schreuder, Carla M. L. van Herpen, Johannes H. W. de Wilt, Ingrid M. E. Desar, Jacky W. J. de Rooy

**Affiliations:** 1 Department of Medical Oncology, Radboud University Medical Centre, 6525 GA Nijmegen, The Netherlands; stefan.ravensteijn@radboudumc.nl (S.G.v.R.); maikel.nederkoorn@radboudumc.nl (M.J.L.N.); tom.wal@radboudumc.nl (T.C.P.W.); yvonne.versleijen-jonkers@radboudumc.nl (Y.M.H.V.-J.); carla.vanherpen@radboudumc.nl (C.M.L.v.H.); 2 Department of Radiotherapy, Radboud University Medical Centre, 6525 GA Nijmegen, The Netherlands; p.braam@radboudumc.nl; 3 Department of Pathology, Radboud University Medical Centre, 6525 GA Nijmegen, The Netherlands; uta.flucke@radboudumc.nl; 4 Department of Surgery, Radboud University Medical Centre, 6525 GA Nijmegen, The Netherlands; han.bonenkamp@radboudumc.nl (J.J.B.); hans.dewilt@radboudumc.nl (J.H.W.d.W.); 5 Department of Orthopedics, Radboud University Medical Centre, 6525 GA Nijmegen, The Netherlands; bart.schreuder@radboudumc.nl; 6 Department of Radiology, Radboud University Medical Centre, 6525 GA Nijmegen, The Netherlands; jacky.derooy@radboudumc.nl

**Keywords:** myxofibrosarcoma, neoadjuvant radiotherapy, magnetic resonance imaging, prognostic factors, local recurrence, tail sign, vascular pedicle, soft tissue sarcoma

## Abstract

**Simple Summary:**

Myxofibrosarcomas (MFS) are malignant soft tissue tumors, frequently located in the extremities. Owing to the infiltrative growth pattern of MFS, neoadjuvant radiotherapy (nRT) is commonly used before surgery to improve local control. Nevertheless, high local recurrence rates are typical in MFS. Data on prognostic factors for poor clinical outcomes are lacking. This retrospective study investigates the prognostic relevance of magnetic resonance imaging (MRI) characteristics before and after nRT in 40 MFS patients. The presence of a vascular pedicle, defined as extra-tumoral vessels at the tumor periphery, was prognostic for both worse disease-free survival (DFS) and overall survival. Additionally, the presence of an infiltrative pattern, referred to as a tail sign, was prognostic for worse DFS. These MRI characteristics could support the identification of patients at risk for poor clinical outcomes after nRT.

**Abstract:**

To improve local control, neoadjuvant radiotherapy (nRT) followed by surgery is the standard of care in myxofibrosarcoma (MFS) because of its infiltrative growth pattern. Nevertheless, local recurrence rates are high. Data on prognostic factors for poor clinical outcomes are lacking. This retrospective study thus investigates the prognostic relevance of magnetic resonance imaging (MRI) characteristics before and after nRT in 40 MFS patients, as well as their association with disease-free survival (DFS) and overall survival (OS). A vascular pedicle, defined as extra-tumoral vessels at the tumor periphery, was observed in 12 patients (30.0%) pre-nRT and remained present post-nRT in all cases. Patients with a vascular pedicle had worse DFS (HR 5.85; 95% CI 1.56–21.90; *p* = 0.009) and OS (HR 9.58; 95% CI 1.91–48.00; *p* = 0.006). An infiltrative growth pattern, referred to as a tail sign, was observed in 22 patients (55.0%) pre-nRT and in 19 patients (47.5%) post-nRT, and was associated with worse DFS post-nRT (HR 6.99; 95% CI 1.39–35.35; *p* = 0.019). The percentage of tumor necrosis estimated by MRI was increased post-nRT, but was not associated with survival outcomes. The presence of a tail sign or vascular pedicle on MRI could support the identification of patients at risk for poor clinical outcomes after nRT.

## 1. Introduction

Myxofibrosarcoma (MFS) is a histological subtype of soft tissue sarcoma (STS), histologically characterized by pleomorphism, myxoid stroma, and curvilinear vasculature [1]. This malignant lesion of mesenchymal origin represents approximately 5% of all sarcoma entities, with an age-standardized incidence rate of 0.19 per 100,000 persons-year in Europe [2]. MFS most commonly presents in the extremities or trunk of patients in the sixth to eighth decades of life, with a particular predilection for the lower limbs [1,3]. The mainstay of treatment for localized primary disease involves surgical resection, commonly applied in conjunction with neoadjuvant radiotherapy (nRT) to optimize local control. MFS is described as a locally aggressive tumor with a distinctive infiltrative pattern of growth, resulting in particularly high rates of local recurrence (LR), ranging from 20 to 60% [3,4,5,6,7,8,9,10,11,12]. Distant metastasis will eventually develop in 20–40% of patients, despite adequate treatment of the primary tumor [3,4,5,6,7,8,9,10,11,12]. Several prognostic factors for LR and overall survival (OS) have been described in MFS, including tumor size and patient age and sex [3,4,5,6,8,11,13]. A recently published large series from the Netherlands comprising 908 MFS patients reported a median OS of 155 months, which was significantly lower in patients who experienced LR (64.0 months) or metastatic disease (34.3 months) [3]. A comprehensive understanding of prognostic factors for patients at risk of poor clinical outcomes despite adequate initial treatment might support the optimization of primary treatment and follow-up schedules.

Recently, neoadjuvant radiotherapy has been replacing adjuvant radiotherapy as the preferred treatment modality, as it enables more accurate definition of the treatment field and thus limits damage to adjacent structures [14,15]. The role of nRT has been investigated in different histological subtypes of STS, but not in MFS, where positive surgical margins and high tumor necrosis have been identified as predictors for worse clinical outcome [16,17,18,19,20,21]. A high percentage of tumor necrosis, histologically assessed, after nRT is the most commonly used surrogate marker for treatment response; however, this has not been validated in STS and small studies present conflicting data [18,19,20,22].

Magnetic resonance imaging (MRI) is the standard of care in MFS diagnosis and restaging after nRT. Specific MRI features at diagnosis, in particular the presence of longitudinal spreading, also referred to as a tail sign, has been recognized as an MRI predictor prognostic for LR and worse OS [5,6,23,24]. The value of post-nRT MRI characteristics as prognostic factors for disease recurrence or OS remains ambiguous and should be explored in further detail. Being able to identify high-risk patients in an early stage might support the development of intensified primary treatment and follow-up strategies for these patients. We thus aim to evaluate the prognostic relevance of MRI characteristics in MFS patients who received nRT.

## 2. Materials and Methods

### 2.1. Study Objectives

The primary objective of this retrospective study was to evaluate the prognostic relevance of pre- and post-nRT MRI characteristics in MFS patients. The secondary objective of this study was to identify factors prognostic for DFS and OS. In addition, the effects of the WHO performance status, surgical margin status, the time interval between nRT and restaging MRI, and the time interval between nRT and surgery on DFS and OS were investigated.

### 2.2. Study Population

Data from patients diagnosed with MFS and treated with nRT in a tertiary sarcoma expertise center in the Netherlands between 2014 and 2022 were retrospectively collected. Histological diagnosis of MFS was confirmed by an experienced sarcoma pathologist (U.E.F.). Patients were included in this study if both pre- and post-nRT MRI data were available. This study was conducted according to the principles of the declaration of Helsinki. Written informed consent was provided by all participating subjects (NCT05373810).

### 2.3. Magnetic Resonance Imaging

Only patients with MRI examinations with the following minimum requirements were included in this study: all MRI studies were performed on a 1.5 Tesla scanner using a protocol including T1-weighted (T1w) and T2-weighted (T2w) sequences with fat saturation, and T1w images with fat saturation after intravenous administration of Gadolinium (Gd). All MRI studies, both pre- and post-nRT, were revised by an experienced musculoskeletal radiologist (J.W.J.d.R.). The musculoskeletal radiologist was blinded to the clinical and histopathological data.

Data on pre- and post-nRT MRI characteristics were systematically collected. Gd enhancement was evaluated on T1w sequences with fat saturation using the grading system proposed by Sambri et al. [5,6]. T1w, T2w, and Gd-enhanced studies were used to evaluate the parameters described below, including the presence of peritumoral edema or intratumoral signs of bleeding, vascular pedicle, necrosis percentage, tumor volume, and tumor size. Myxoid matrix content was recognized as the presence of high signal on fluid-sensitive sequences (T2w), slightly less than the signal of water [5,6]. A vascular pedicle was defined as abnormal tortuous feeding extra-tumoral vessels, whether or not coalescing as a clump, at one site along the lesion periphery. This peri-tumoral neo-angiogenesis can be identified as prominent flow voids (i.e., signal loss) [25,26]. The percentage of tumor necrosis was evaluated using T1w sequences with fat saturation after Gd contrast enhancement on a semi-quantitative base (<50.0% or ≥50.0%). Tumor size was measured as the largest diameter of the mass in any direction of the imaging plane. Tumor shape was classified as monolobular, lobular, or polylobulated. Tumor location was recorded as superficial (above the fascia) or deep (below the fascia). The number of anatomical compartments involved was registered, as was the tumor demarcation (unsharp, moderately sharp, sharp). The presence of an infiltrative growth pattern, referred to as a tail sign, was determined and differentiated from peritumoral edema by its enhancement on post-contrast images. A tail sign was deemed present if the tail was at least 10 mm in length and 2 mm in width [23,27].

### 2.4. Clinical and Histopathological Data

Clinical data were retrieved from a prospective clinical registry database and included patient age and BMI at diagnosis, sex, tumor site, WHO performance status, date and dose schedule of nRT, timing of MRI studies, the occurrence of LR, time interval between surgery and LR, treatment for LR, the occurrence of metastasis, time interval between surgery and metastasis, treatment for metastasis, and date of death or last follow-up. Histopathological data included tumor grading and classification of surgical margins according to the guidelines of the American Joint Committee on Cancer (R0: tumor-free margin, R1: microscopic positive margin, and R2: macroscopic positive margin) [28].

### 2.5. Statistical Analysis

Descriptive statistics were used to describe baseline patient characteristics. Mean or median values were described as applicable. Continuous variables were compared through *t*-test or Mann–Whitney U test. To compare categorical variables, McNemar-(Bowker) tests were used. Values were considered significant with a *p*-value ≤ 0.05. Univariate survival analyses were performed according to the Kaplan–Meier method. Multivariable Cox regression was performed using forward Wald selection. Variables that revealed a *p*-value ≤ 0.10 were included in the multivariable model. Statistical analyses were performed using IBM SPSS statistics 25 (version 3.6.2) and RStudio (version 1.1.463).

## 3. Results

### 3.1. Patient Characteristics

The total cohort comprised 40 MFS patients. Patient characteristics are depicted in Table 1. The majority of primary MFS tumors were located in the lower extremities (77.5%). Most lesions were located below the fascia (70.0%) and were of high histologic grade (92.5%). The most frequently applied nRT dose was 50 Gray (Gy) in 25 fractions (25 × 2 Gy) (87.5%). Four patients (10.0%) received 45 Gy in 15 fractions (15 × 3 Gy) and one patient (2.5%) received 25 Gy in 5 fractions (5 × 5 Gy) based on the physicians’ choice. All patients with MFS involving the extremities who received nRT underwent limb-sparing resection. The median time intervals between nRT and restaging MRI or surgery were 32 (range 12–61) days and 51 (range 26–117) days, respectively. Negative surgical margins (R0) were reported in 90.0% of cases. Microscopic-positive surgical margins (R1) were described in 5.0% of patients and 5.0% of cases had macroscopic-positive surgical margins (R2).

### 3.2. Neoadjuvant Radiotherapy Induced Changes in MRI Characteristics

Pre-nRT MRI studies were predominantly performed at the primary referral center and, therefore, there was a large variance in MRI devices and pulse sequences. An overview of the observed pre- and post-nRT MRI characteristics is provided in Table 2. The median tumor diameter (81 mm pre-nRT versus (vs.) 92 mm post-nRT, *p* = 0.002) and median tumor volume (224 cm^3^ pre-nRT vs. 298 cm^3^ post-nRT, *p* = 0.008) were significantly increased post-nRT. The number of patients with a tumor necrosis percentage ≥50.0% significantly increased post-nRT (10.0% pre-nRT vs. 45.0% post-nRT, *p* = 0.046). Three patients showed no signs of tumor necrosis post-nRT. Furthermore, there was a significant increase in the number of cases that showed tumor bleeding (47.5% pre-nRT vs. 67.5% post-nRT, *p* = 0.021). Peritumoral edema was present in 97.5% of cases pre-nRT and in 100.0% of cases post-nRT (*p* = 1.000).

**Figure 1:**
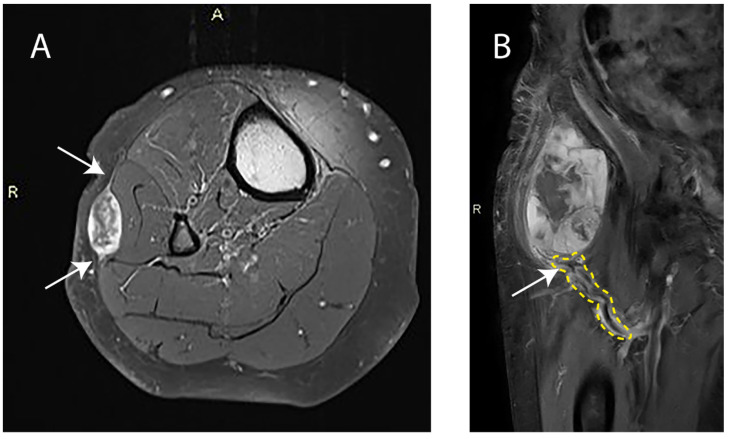
(**A**) Axial-contrast-enhanced T1-weighted image with fat saturation of a right lower leg with a superficial myxofibrosarcoma. Arrows indicate the tail sign. A: Anterior orientation; R: Right side orientation. (**B**) Coronal-contrast-enhanced T1-weighted image of a right upper leg with a deep myxofibrosarcoma. The area outlined in yellow highlights the pathologic tumor-feeding vasculature and the arrow indicates the vascular pedicle. R: Right side orientation.

A vascular pedicle, defined as abnormal tortuous feeding extra-tumoral vessels, whether or not coalescing as a clump, at one site along the lesion periphery (Figure 1), was observed in 12 patients pre-nRT (30.0%) and remained present post-nRT in all cases. No new cases with a vascular pedicle were identified on post-nRT MRI. When a vascular pedicle was present, 1 out of 12 patients had positive surgical margins, categorized as R1 or R2. In the absence of a vascular pedicle, 3 out of 28 patients had positive surgical margins (*p* = 0.824). An infiltrative pattern with extensions of ≥10 mm in length and ≥2 mm in width (Figure 1), also referred to as a tail sign, was present in 22 cases pre-nRT (55.0%) and in 19 cases (47.5%) post-nRT (*p* = 0.453). All lesions with a tail sign observed post-nRT were already observed pre-nRT. No new cases with a tail-sign-containing lesion were identified post-nRT. Furthermore, no significant changes were observed in tail sign length (*p* = 0.134) or tail sign width (*p* = 0.201) post-nRT. In the presence of a tail sign post-nRT, 4 out of 19 patients had positive surgical margins. In its absence, 0 out of 21 patients had positive surgical margins (*p* = 0.027).

### 3.3. Prognostic Relevance of Post-nRT MRI Characteristics

**Figure 2:**
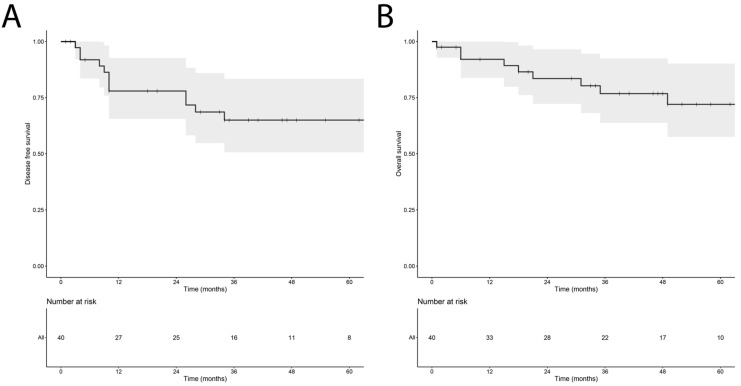
Kaplan–Meier estimate reflecting the disease-free survival (**A**) and overall survival (**B**) of the cohort of 40 myxofibrosarcoma patients, including the 95% confidence interval.

The median follow-up after surgery was 44 (range 2–103) months. Throughout the follow-up period, LR occurred in four patients (10.0%) and nine patients (22.5%) developed distant metastases. Nine patients (22.5%) deceased, of whom seven passed away from disease-related causes. Figure 2 depicts the Kaplan–Meier estimates for DFS and OS for the entire patient cohort over time. Median DFS and OS were not reached during the follow-up period.

**Figure 3:**
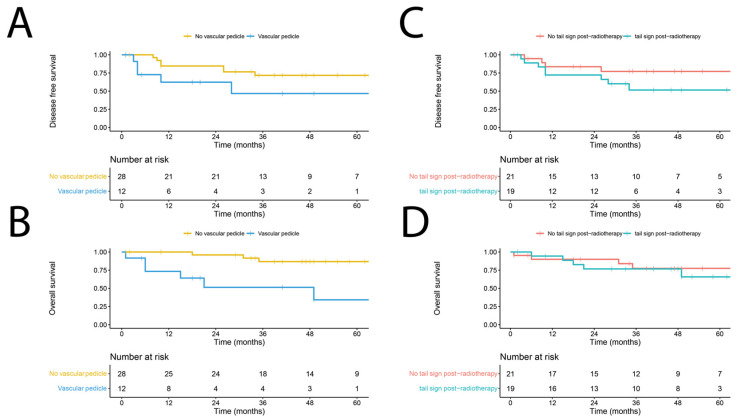
Kaplan–Meier estimate reflecting the disease-free survival (DFS) (**A**) and overall survival (OS) (**B**) of myxofibrosarcoma in the presence or absence of a vascular pedicle on MRI, and DFS (**C**) and OS (**D**) of patients in the presence or absence of a tail sign on post-neoadjuvant radiotherapy MRI.

An exploratory multivariable Cox regression analysis was performed using forward Wald selection on the MRI characteristics depicted in Table 2, with the inclusion of WHO performance status, surgical margin, tumor depth, myxoid type on MRI, and Gd enhancement. The presence of a vascular pedicle (hazard ratio (HR) 5.85; 95% confidence interval (CI) 1.56–21.90; *p* = 0.009) and a tail sign (HR 6.99; 95% CI 1.39–35.35; *p* = 0.019) post-nRT were associated with worse DFS. The presence of a vascular pedicle post-nRT was the sole factor associated with worse OS (HR 9.58; 95% CI 1.91–48.00; *p* = 0.006). Figure 3 depicts the Kaplan–Meier estimates for DFS and OS in relation to the presence of a vascular pedicle or tail sign post-nRT. None of the other remaining patient or MRI characteristics post-nRT demonstrated a significant association with DFS or OS.

## 4. Discussion

In this large retrospective MFS series investigating the prognostic relevance of MRI characteristics in patients who received nRT, the presence of a vascular pedicle or tail sign on post-nRT MRI was prognostic for worse survival outcomes. The presence of a vascular pedicle on post-nRT MRI was prognostic for both worse DFS and OS, whereas the post-nRT presence of a tail-sign-containing lesion was exclusively prognostic for worse DFS. These MRI characteristics could serve as prognostic biomarkers to support the non-invasive identification of patients at risk of worse clinical outcomes in an early stage.

We identified a vascular pedicle in 30.0% of cases post-nRT, of whom 41.7% eventually developed LR (*n* = 1, 8.3%) or distant metastasis (*n* = 4, 33.3%). In the absence of a vascular pedicle, only 25.0% of cases developed LR (*n* = 2, 7.1%), distant metastasis (*n* = 4, 14.3%), or both (*n* = 1, 3.6%). We found no relationship between the presence of a vascular pedicle and surgical margin status. Furthermore, we observed no changes in the presence of a vascular pedicle between pre- and post-nRT MRI. This suggests that the presence of a vascular pedicle on pre-nRT MRI has similar prognostic value to its presence on post-nRT MRI. While the presence of a vascular pedicle has been reported in different histological subtypes of STS, this feature has not yet been described in MFS. Ledoux et al. identified abnormal peritumor vascularization in 17% of STS patients using MRI in a cohort of 157 STS cases, including 24 MFS patients. The authors found that peritumoral flow voids might be associated with a higher risk of metastatic relapse and poorer OS. They hypothesize that abnormal vascularization might favor the occurrence of hematogenous metastasis through the formation of endovascular thrombi of tumor cells [26,29]. In the current study, we report that patients with a vascular pedicle, defined as either a real clump of peri-tumoral vessels or as abnormal feeding peri-tumoral vascularization, are at increased risk of developing recurrent disease after nRT. As most patients with disease recurrence had metastatic disease, we suggest that the vascular pedicle could be involved in the hematogenic spread of cancer cells. Future studies using dynamic-contrast-enhanced (DCE) MRI may quantify tumor-related perfusion and further characterize peritumoral microstructures. Further research is needed to investigate the optimal surgical strategy regarding a vascular pedicle. One might hypothesize that, when a surgeon is informed about the presence of a vascular pedicle, this should also be resected to further prevent hematogenic spread of cancer cells. The same holds for considerations regarding adjuvant systemic therapy.

The presence of a tail sign on MRI is the most recognized feature prognostic for worse DFS in MFS. In this study, we report the presence of a tail sign in 55.0% of cases pre-nRT and in 47.5% of cases post-nRT. This finding is in line with previous studies describing the presence of tail-like lesions in MFS. Lefkowitz et al. reported the presence of a tail sign in 64.0–77.0% of cases on MRI in a cohort of 44 MFS patients, which was associated with worse DFS, but not OS. We found a relationship between the presence of a tail sign and positive surgical margins. This finding could be consistent with the worse DFS observed in the presence of a tail sign. The presence of a tail sign has also been reported as an independent adverse prognostic factor for local control and metastasis-free survival after surgery in a cohort of 89 STS [30]. The histological effect of nRT on tail-like lesions was investigated in a cohort of 18 STS cases, comprising 8 MFS patients and 10 cases of undifferentiated pleomorphic sarcoma. Viable tumor cells remained present after treatment with nRT in 8/18 cases, of whom three patients developed locally recurrent disease [31]. We found no significant changes after nRT in the presence, length, or width of the tail sign. The complete disappearance or shrinkage of tail-like lesions after neoadjuvant treatment has been reported in 33.3% of cases in a cohort of 36 STS patients, including 13 MFS cases, but did not impact the oncological outcomes [32].

Tumor necrosis after nRT is the most studied prognostic factor in sarcoma. We found no correlation between the tumor necrosis percentage estimated by MRI and survival outcomes. Quantification of the tumor necrosis percentage by means of MRI is challenging owing to tissue heterogeneity, inter-observer variability, and a wide variety of pulse sequences. Diffusion-weighted imaging (DWI) MRI is not yet implemented in standard care, while it has the best capacity to quantify tumor necrosis. In the case of histopathological assessment, the estimation of the tumor necrosis percentage is subject to the selection of microscopic fields and sampling heterogeneity. Furthermore, within the European Organisation for Research and Treatment of Cancer (EORTC) scoring system for necrosis, cutoff percentages have been arbitrarily chosen [33]. Not surprisingly, data on the tumor necrosis percentage in STS in relation to clinical outcomes are contradictory [18,20]. The data obtained in this study support the conclusion that, in contrast to bone sarcomas, tumor necrosis has no clear prognostic value in MFS [19,34].

The current integration of nRT into the standard of care of MFS is hindered by the increased incidence of wound healing complications compared with adjuvant radiotherapy. Because of the lack of literature on the nRT-to-surgery time interval and its prognostic value in STS, we explored the influence of the time interval between the end of nRT and surgery on DFS and OS in MFS, and could not identify a clear cut-off. Currently, the EORTC recommends that imaging should not be performed earlier than 4 weeks after nRT [35]. Collier et al. reported, in a large retrospective database study investigating the nRT-surgery interval in STS, that a delay in surgery of up to 120 days after nRT was not associated with worse survival [36]. An interval of six weeks between the end of nRT and surgery was associated with fewer wound complications in another study [37]. The time interval between the end of nRT and surgery in STS warrants further investigation to minimize wound complications after nRT without affecting survival outcomes.

The next step after our retrospective study should be to perform a prospective trial using multiparametric MRI in a cohort of MFS patients to assess tumor cellularity and vascularization after nRT. Multiparametric MRI should be performed at multiple time points between the end of nRT and planned surgery using the recommendations of the EORTC Soft Tissue and Bone Sarcoma Imaging Group [35,38,39]. In the past, several adjuvant chemotherapy trials in different histological subtypes of STS failed to improve the survival of heterogeneous cohorts of STS patients. This has been attributed to the lack of selection of real high-risk patients with chemotherapy-sensitive STS histiotypes [40]. The use of MRI characteristics as prognostic biomarkers to select high-risk patients and to individualize further follow-up or treatment should be investigated in further detail.

## 5. Conclusions

The presence of a tail sign after nRT is prognostic for worse DFS and the presence of a vascular pedicle is prognostic for both worse DFS and OS, both pre- and post-nRT. These MRI characteristics could serve as biomarkers to support the identification of MFS patients at risk for dismal clinical outcomes in an early stage.

## Figures and Tables

**Table 1:** Baseline patient and MRI characteristics. All MRI characteristics reflected in this table were described based on pre-nRT imaging.

Number of Patients = 40	
Gender *n* (%)	
Male Female	23 (57.5) 17 (42.5)
Median BMI at diagnosis (range)	25.2 (19.8–45.3)
Median age in years at diagnosis (range)	67 (48–86)
Primary tumor site *n* (%)	
Upper leg Lower leg Lower arm Shoulder Knee Upper arm Abdominal wall Thoracic wall	24 (60.0) 7 (17.5) 2 (5.0) 2 (5.0) 2 (5.0) 1 (2.5) 1 (2.5) 1 (2.5)
WHO performance status (%)	
0 1	24 (60.0) 16 (40.0)
Radiotherapy dosing schedule (Gy) *n* (%)	
50 Gy in 25 fractions 45 Gy in 15 fractions 25 Gy in 5 fractions	35 (87.5) 4 (10.0) 1 (2.5)
Surgical margin	
R0 R1 R2	36 (90.0) 2 (5.0) 2 (5.0)
Tumor grading *n* (%)	
High grade Low grade Unknown	37 (92.5) 2 (5.0) 1 (2.5)
Tumor depth on MRI, *n* (%)	
Deep Superficial	28 (70.0) 12 (30.0)
Myxoid type based on MRI, *n* (%)	
Grade 0 Grade 1 Grade 2 Grade 3	5 (12.5) 9 (22.5) 21 (52.5) 5 (12.5)
Gadolinium enhancement on MRI, *n* (%)	
Grade 0 Grade 1 Grade 2 Grade 3	6 (15.0) 8 (20.0) 11 (27.5) 15 (37.5)

**Table 2:** Pre- and post-neoadjuvant radiotherapy MRI characteristics.

MRI Characteristic	Pre-Radiotherapy MRI	Post-Radiotherapy MRI	*p*-Value
Median tumor diameter in mm (range)	81 (16–231)	92 (0–273)	0.002
Median tumor volume in cm^3^ (range)	224 (2.6–3114.3)	298 (1.2–4440.8)	0.008
Tail sign present *n* (%)	22 (55.0)	19 (47.5)	0.453
Median tail sign length in mm (range)	30 (10–60)	27(12–91)	0.134
Median tail sign width in mm (range)	5 (2–11)	6 (2–21)	0.201
Vascular pedicle present *n* (%)			1.000
Yes No	12 (30.0) 28 (70.0)	12 (30.0) 28 (70.0)	
Necrosis % *n* (%)			0.046
<50.0% ≥50.0% Unassessable	34 (85.0) 4 (10.0) 2 (5.0)	21 (52.5) 18 (45.0) 1 (2.5)	
Edema *n* (%)			1.000
Present Not present	39 (97.5) 1 (2.5)	40 (100.0) 0 (0.0)	
Tumor demarcation *n* (%)			0.317
Unsharp Moderately sharp Sharp Unassessable	5 (12.5) 6 (15.0) 29 (72.5)	3 (7.5) 7 (17.5) 29 (72.5) 1 (2.5)	
Neurovascular relation *n* (%)			0.135
No relation Close relation Encasement	32 (80.0) 6 (15.0) 2 (5.0)	36 (90.0) 3 (7.5) 1 (2.5)	
Number of compartments *n* (%)			1.000
1 2/3	30 (75.0) 10 (25.0)	29 (72.5) 11 (27.5)	
Tumor bleeding *n* (%)			0.021
Present Not present	19 (47.5) 21 (52.5)	27 (67.5) 13 (32.5)	
Tumor shape *n* (%)			0.607
Homogenous Lobular Polylobular Multi-locular Unassessable	2 (5.0) 5 (12.5) 32 (80.0) 1 (2.5)	3 (7.5) 5 (12.5) 30 (75.0) 1 (2.5) 1 (2.5)	

## Data Availability

The data presented in this study are available upon reasonable request from the corresponding author.
